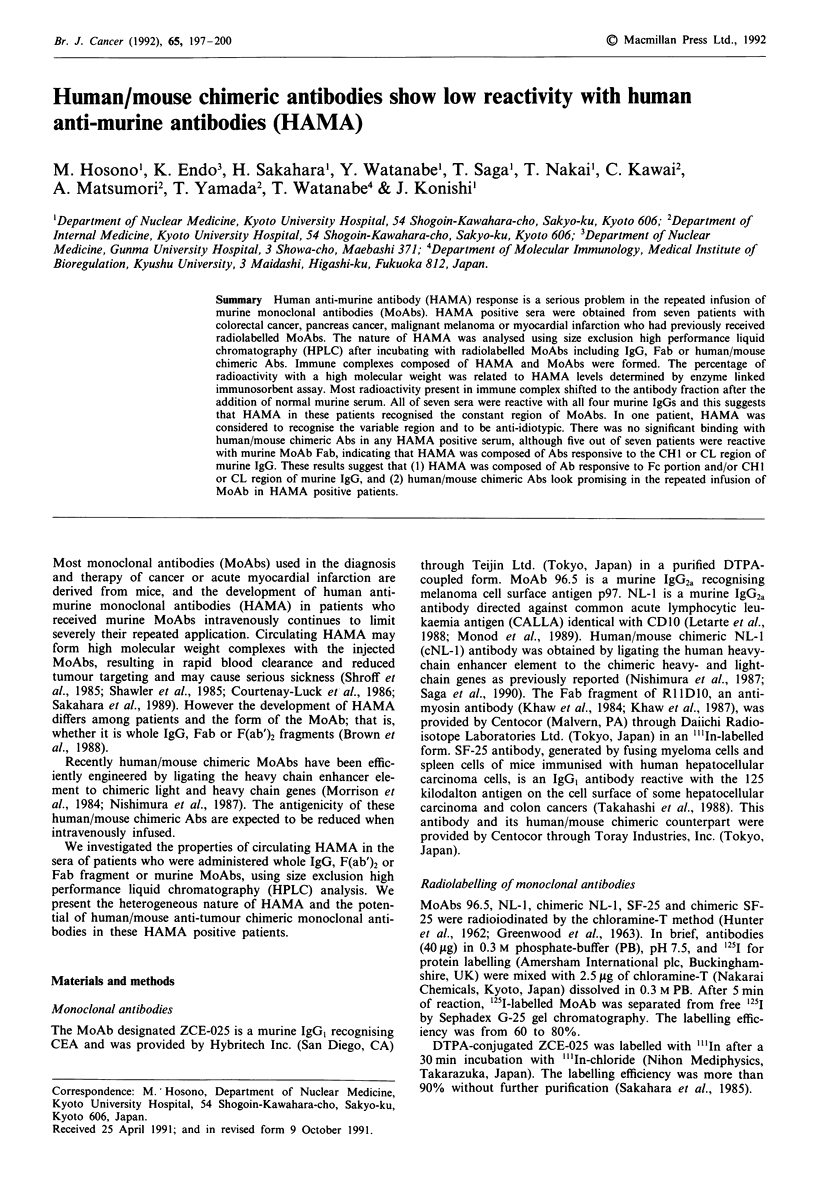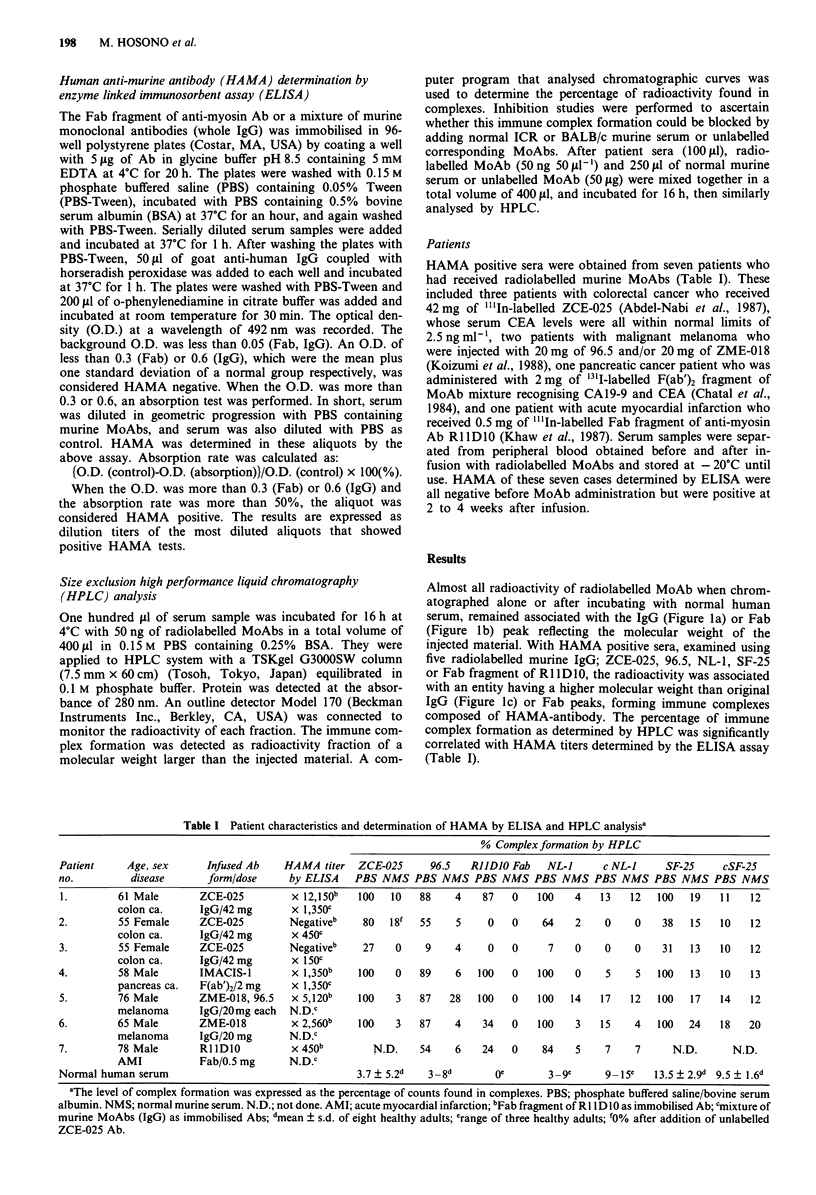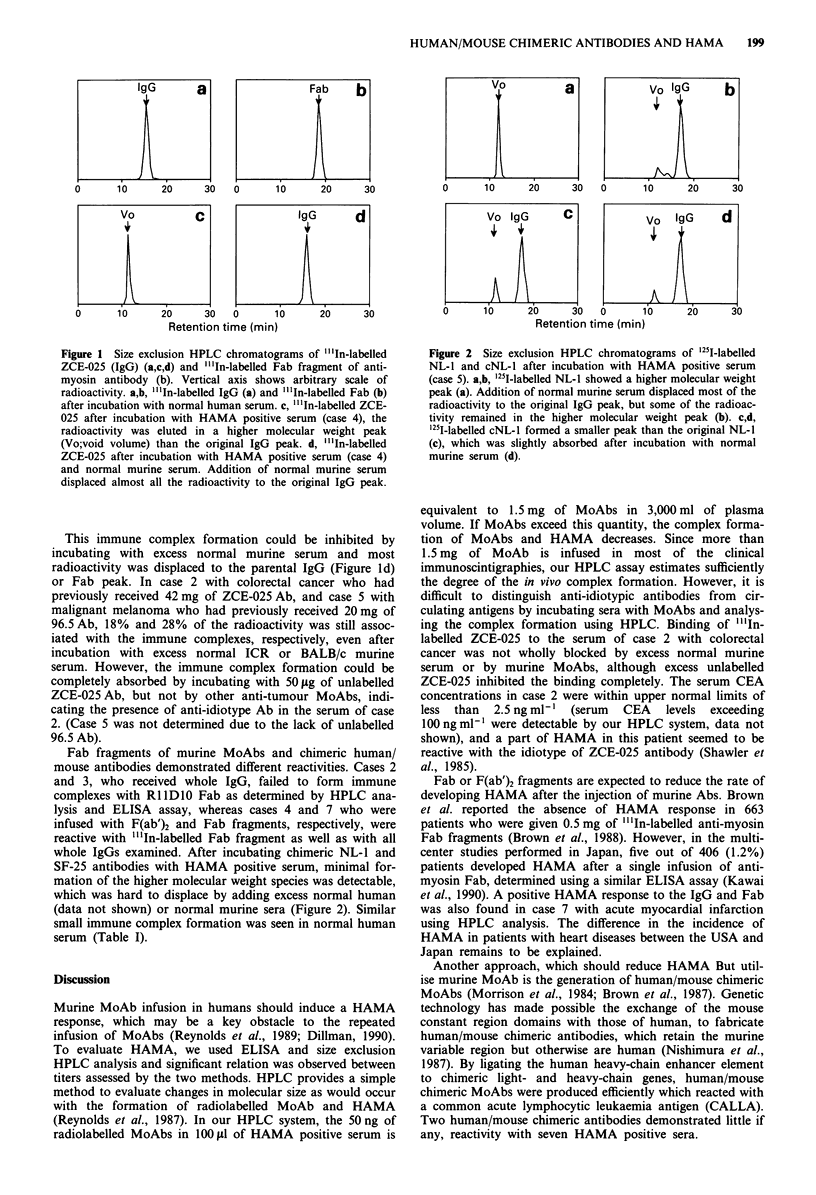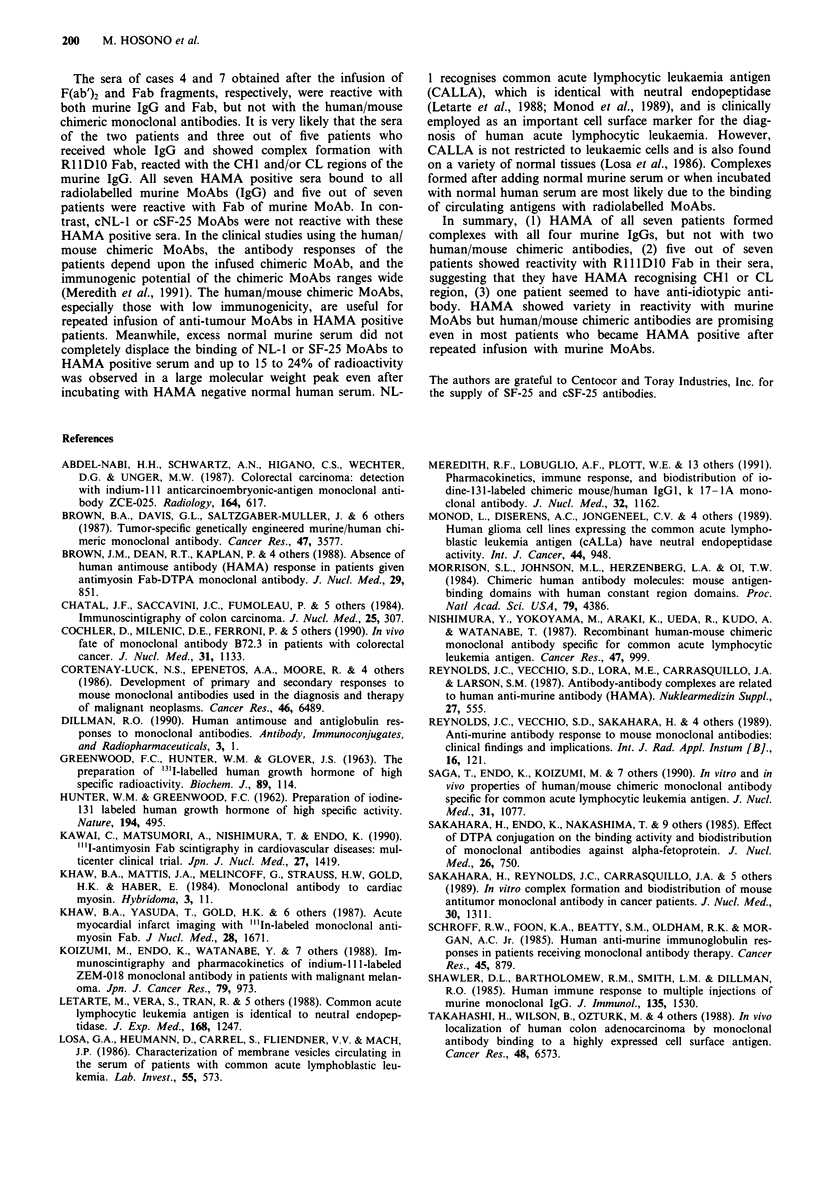# Human/mouse chimeric antibodies show low reactivity with human anti-murine antibodies (HAMA).

**DOI:** 10.1038/bjc.1992.41

**Published:** 1992-02

**Authors:** M. Hosono, K. Endo, H. Sakahara, Y. Watanabe, T. Saga, T. Nakai, C. Kawai, A. Matsumori, T. Yamada, T. Watanabe

**Affiliations:** Department of Nuclear Medicine, Kyoto University Hospital, Japan.

## Abstract

Human anti-murine antibody (HAMA) response is a serious problem in the repeated infusion of murine monoclonal antibodies (MoAbs). HAMA positive sera were obtained from seven patients with colorectal cancer, pancreas cancer, malignant melanoma or myocardial infarction who had previously received radiolabelled MoAbs. The nature of HAMA was analysed using size exclusion high performance liquid chromatography (HPLC) after incubating with radiolabelled MoAbs including IgG, Fab or human/mouse chimeric Abs. Immune complexes composed of HAMA and MoAbs were formed. The percentage of radioactivity with a high molecular weight was related to HAMA levels determined by enzyme linked immunosorbent assay. Most radioactivity present in immune complex shifted to the antibody fraction after the addition of normal murine serum. All of seven sera were reactive with all four murine IgGs and this suggests that HAMA in these patients recognised the constant region of MoAbs. In one patient, HAMA was considered to recognise the variable region and to be anti-idiotypic. There was no significant binding with human/mouse chimeric Abs in any HAMA positive serum, although five out of seven patients were reactive with murine MoAb Fab, indicating that HAMA was composed of Abs responsive to the CH1 or CL region of murine IgG. These results suggest that (1) HAMA was composed of Ab responsive to Fc portion and/or CH1 or CL region of murine IgG, and (2) human/mouse chimeric Abs look promising in the repeated infusion of MoAb in HAMA positive patients.


Br. J. Cancer (1992), 65, 197–200 © Macmillan Press Ltd., 1992

### Human/mouse chimeric antibodies show low reactivity with human anti-murine antibodies (HAMA)

M. Hosono[1], K. Endo[3], H. Sakahara[1], Y. Watanabe[1], T. Saga[1], T. Nakai[1], C. Kawai[2], A. Matsumori[2], T. Yamada[2], T. Watanabe[4] & J. Konishi[1]

[1]*Department of Nuclear Medicine, Kyoto University Hospital, 54 Shogoin-Kawahara-cho, Sakyo-ku, Kyoto 606;* [2]*Department of Internal Medicine, Kyoto University Hospital, 54 Shogoin-Kawahara-cho, Sakyo-ku, Kyoto 606;* [3]*Department of Nuclear Medicine, Gunma University Hospital, 3 Showa-cho, Maebashi 371;* [4]*Department of Molecular Immunology, Medical Institute of Bioregulation, Kyushu University, 3 Maidashi, Higashi-ku, Fukuoka 812, Japan.*

**Summary** Human anti-murine antibody (HAMA) response is a serious problem in the repeated infusion of murine monoclonal antibodies (MoAbs). HAMA positive sera were obtained from seven patients with colorectal cancer, pancreas cancer, malignant melanoma or myocardial infarction who had previously received radiolabelled MoAbs. The nature of HAMA was analysed using size exclusion high performance liquid chromatography (HPLC) after incubating with radiolabelled MoAbs including IgG, Fab or human/mouse chimeric Abs. Immune complexes composed of HAMA and MoAbs were formed. The percentage of radioactivity with a high molecular weight was related to HAMA levels determined by enzyme linked immunosorbent assay. Most radioactivity present in immune complex shifted to the antibody fraction after the addition of normal murine serum. All of seven sera were reactive with all four murine IgGs and this suggests that HAMA in these patients recognised the constant region of MoAbs. In one patient, HAMA was considered to recognise the variable region and to be anti-idiotypic. There was no significant binding with human/mouse chimeric Abs in any HAMA positive serum, although five out of seven patients were reactive with murine MoAb Fab, indicating that HAMA was composed of Abs responsive to the CH1 or CL region of murine IgG. These results suggest that (1) HAMA was composed of Ab responsive to Fc portion and/or CH1 or CL region of murine IgG, and (2) human/mouse chimeric Abs look promising in the repeated infusion of MoAb in HAMA positive patients.

Most monoclonal antibodies (MoAbs) used in the diagnosis and therapy of cancer or acute myocardial infarction are derived from mice, and the development of human anti-murine monoclonal antibodies (HAMA) in patients who received murine MoAbs intravenously continues to limit severely their repeated application. Circulating HAMA may form high molecular weight complexes with the injected MoAbs, resulting in rapid blood clearance and reduced tumour targeting and may cause serious sickness (Shroff *et al.*, 1985; Shawler *et al.*, 1985; Courtenay-Luck *et al.*, 1986; Sakahara *et al.*, 1989). However the development of HAMA differs among patients and the form of the MoAb; that is, whether it is whole IgG, Fab or F(ab')$_2$ fragments (Brown *et al.*, 1988).

Recently human/mouse chimeric MoAbs have been efficiently engineered by ligating the heavy chain enhancer element to chimeric light and heavy chain genes (Morrison *et al.*, 1984; Nishimura *et al.*, 1987). The antigenicity of these human/mouse chimeric Abs are expected to be reduced when intravenously infused.

We investigated the properties of circulating HAMA in the sera of patients who were administered whole IgG, F(ab')$_2$ or Fab fragment or murine MoAbs, using size exclusion high performance liquid chromatography (HPLC) analysis. We present the heterogeneous nature of HAMA and the potential of human/mouse anti-tumour chimeric monoclonal antibodies in these HAMA positive patients.

### Materials and methods

### *Monoclonal antibodies*

The MoAb designated ZCE-025 is a murine IgG$_1$ recognising CEA and was provided by Hybritech Inc. (San Diego, CA) through Teijin Ltd. (Tokyo, Japan) in a purified DTPA-coupled form. MoAb 96.5 is a murine IgG$_{2a}$ recognising melanoma cell surface antigen p97. NL-1 is a murine IgG$_{2a}$ antibody directed against common acute lymphocytic leukaemia antigen (CALLA) identical with CD10 (Letarte *et al.*, 1988; Monod *et al.*, 1989). Human/mouse chimeric NL-1 (cNL-1) antibody was obtained by ligating the human heavy-chain enhancer element to the chimeric heavy- and light-chain genes as previously reported (Nishimura *et al.*, 1987; Saga *et al.*, 1990). The Fab fragment of R11D10, an anti-myosin antibody (Khaw *et al.*, 1984; Khaw *et al.*, 1987), was provided by Centocor (Malvern, PA) through Daiichi Radioisotope Laboratories Ltd. (Tokyo, Japan) in an $^{111}$In-labelled form. SF-25 antibody, generated by fusing myeloma cells and spleen cells of mice immunised with human hepatocellular carcinoma cells, is an IgG$_1$ antibody reactive with the 125 kilodalton antigen on the cell surface of some hepatocellular carcinoma and colon cancers (Takahashi *et al.*, 1988). This antibody and its human/mouse chimeric counterpart were provided by Centocor through Toray Industries, Inc. (Tokyo, Japan).

### *Radiolabelling of monoclonal antibodies*

MoAbs 96.5, NL-1, chimeric NL-1, SF-25 and chimeric SF-25 were radioiodinated by the chloramine-T method (Hunter *et al.*, 1962; Greenwood *et al.*, 1963). In brief, antibodies (40 µg) in 0.3 M phosphate-buffer (PB), pH 7.5, and $^{125}$I for protein labelling (Amersham International plc, Buckinghamshire, UK) were mixed with 2.5 µg of chloramine-T (Nakarai Chemicals, Kyoto, Japan) dissolved in 0.3 M PB. After 5 min of reaction, $^{125}$I-labelled MoAb was separated from free $^{125}$I by Sephadex G-25 gel chromatography. The labelling efficiency was from 60 to 80%.

DTPA-conjugated ZCE-025 was labelled with $^{111}$In after a 30 min incubation with $^{111}$In-chloride (Nihon Mediphysics, Takarazuka, Japan). The labelling efficiency was more than 90% without further purification (Sakahara *et al.*, 1985).

Correspondence: M. Hosono, Department of Nuclear Medicine, Kyoto University Hospital, 54 Shogoin-Kawahara-cho, Sakyo-ku, Kyoto 606, Japan.
Received 25 April 1991; and in revised form 9 October 1991.

### *Human anti-murine antibody (HAMA) determination by enzyme linked immunosorbent assay (ELISA)*

The Fab fragment of anti-myosin Ab or a mixture of murine monoclonal antibodies (whole IgG) was immobilised in 96-well polystyrene plates (Costar, MA, USA) by coating a well with 5 μg of Ab in glycine buffer pH 8.5 containing 5 mM EDTA at 4°C for 20 h. The plates were washed with 0.15 M phosphate buffered saline (PBS) containing 0.05% Tween (PBS-Tween), incubated with PBS containing 0.5% bovine serum albumin (BSA) at 37°C for an hour, and again washed with PBS-Tween. Serially diluted serum samples were added and incubated at 37°C for 1 h. After washing the plates with PBS-Tween, 50 μl of goat anti-human IgG coupled with horseradish peroxidase was added to each well and incubated at 37°C for 1 h. The plates were washed with PBS-Tween and 200 μl of o-phenylenediamine in citrate buffer was added and incubated at room temperature for 30 min. The optical density (O.D.) at a wavelength of 492 nm was recorded. The background O.D. was less than 0.05 (Fab, IgG). An O.D. of less than 0.3 (Fab) or 0.6 (IgG), which were the mean plus one standard deviation of a normal group respectively, was considered HAMA negative. When the O.D. was more than 0.3 or 0.6, an absorption test was performed. In short, serum was diluted in geometric progression with PBS containing murine MoAbs, and serum was also diluted with PBS as control. HAMA was determined in these aliquots by the above assay. Absorption rate was calculated as:

{O.D. (control)-O.D. (absorption)}/O.D. (control) × 100(%).

When the O.D. was more than 0.3 (Fab) or 0.6 (IgG) and the absorption rate was more than 50%, the aliquot was considered HAMA positive. The results are expressed as dilution titers of the most diluted aliquots that showed positive HAMA tests.

### *Size exclusion high performance liquid chromatography (HPLC) analysis*

One hundred μl of serum sample was incubated for 16 h at 4°C with 50 ng of radiolabelled MoAbs in a total volume of 400 μl in 0.15 M PBS containing 0.25% BSA. They were applied to HPLC system with a TSKgel G3000SW column (7.5 mm × 60 cm) (Tosoh, Tokyo, Japan) equilibrated in 0.1 M phosphate buffer. Protein was detected at the absorbance of 280 nm. An outline detector Model 170 (Beckman Instruments Inc., Berkley, CA, USA) was connected to monitor the radioactivity of each fraction. The immune complex formation was detected as radioactivity fraction of a molecular weight larger than the injected material. A computer program that analysed chromatographic curves was used to determine the percentage of radioactivity found in complexes. Inhibition studies were performed to ascertain whether this immune complex formation could be blocked by adding normal ICR or BALB/c murine serum or unlabelled corresponding MoAbs. After patient sera (100 μl), radiolabelled MoAb (50 ng 50 $\mu l^{-1}$) and 250 μl of normal murine serum or unlabelled MoAb (50 μg) were mixed together in a total volume of 400 μl, and incubated for 16 h, then similarly analysed by HPLC.

### *Patients*

HAMA positive sera were obtained from seven patients who had received radiolabelled murine MoAbs (Table I). These included three patients with colorectal cancer who received 42 mg of $^{111}$In-labelled ZCE-025 (Abdel-Nabi *et al.*, 1987), whose serum CEA levels were all within normal limits of 2.5 ng $ml^{-1}$, two patients with malignant melanoma who were injected with 20 mg of 96.5 and/or 20 mg of ZME-018 (Koizumi *et al.*, 1988), one pancreatic cancer patient who was administered with 2 mg of $^{131}$I-labelled F(ab′)$_2$ fragment of MoAb mixture recognising CA19-9 and CEA (Chatal *et al.*, 1984), and one patient with acute myocardial infarction who received 0.5 mg of $^{111}$In-labelled Fab fragment of anti-myosin Ab R11D10 (Khaw *et al.*, 1987). Serum samples were separated from peripheral blood obtained before and after infusion with radiolabelled MoAbs and stored at −20°C until use. HAMA of these seven cases determined by ELISA were all negative before MoAb administration but were positive at 2 to 4 weeks after infusion.

### Results

Almost all radioactivity of radiolabelled MoAb when chromatographed alone or after incubating with normal human serum, remained associated with the IgG (Figure 1a) or Fab (Figure 1b) peak reflecting the molecular weight of the injected material. With HAMA positive sera, examined using five radiolabelled murine IgG; ZCE-025, 96.5, NL-1, SF-25 or Fab fragment of R11D10, the radioactivity was associated with an entity having a higher molecular weight than original IgG (Figure 1c) or Fab peaks, forming immune complexes composed of HAMA-antibody. The percentage of immune complex formation as determined by HPLC was significantly correlated with HAMA titers determined by the ELISA assay (Table I).

**Table I** Patient characteristics and determination of HAMA by ELISA and HPLC analysis[a]

| | | | | *% Complex formation by HPLC* | | | | | | | | | | | | | |
|---|---|---|---|---|---|---|---|---|---|---|---|---|---|---|---|---|---|
| | | | | *ZCE-025* | | *96.5* | | *R11D10 Fab* | | *NL-1* | | *c NL-1* | | *SF-25* | | *cSF-25* | |
| *Patient no.* | *Age, sex disease* | *Infused Ab form/dose* | *HAMA titer by ELISA* | *PBS* | *NMS* | *PBS* | *NMS* | *PBS* | *NMS* | *PBS* | *NMS* | *PBS* | *NMS* | *PBS* | *NMS* | *PBS* | *NMS* |
| 1. | 61 Male colon ca. | ZCE-025 IgG/42 mg | × 12,150[b] × 1,350[c] | 100 | 10 | 88 | 4 | 87 | 0 | 100 | 4 | 13 | 12 | 100 | 19 | 11 | 12 |
| 2. | 55 Female colon ca. | ZCE-025 IgG/42 mg | Negative[b] × 450[c] | 80 | 18[f] | 55 | 5 | 0 | 0 | 64 | 2 | 0 | 0 | 38 | 15 | 10 | 12 |
| 3. | 55 Female colon ca. | ZCE-025 IgG/42 mg | Negative[b] × 150[c] | 27 | 0 | 9 | 4 | 0 | 0 | 7 | 0 | 0 | 0 | 31 | 13 | 10 | 12 |
| 4. | 58 Male pancreas ca. | IMACIS-1 F(ab′)$_2$/2 mg | × 1,350[b] × 1,350[c] | 100 | 0 | 89 | 6 | 100 | 0 | 100 | 0 | 5 | 5 | 100 | 13 | 10 | 13 |
| 5. | 76 Male melanoma | ZME-018, 96.5 IgG/20 mg each | × 5,120[b] N.D.[c] | 100 | 3 | 87 | 28 | 100 | 0 | 100 | 14 | 17 | 12 | 100 | 17 | 14 | 12 |
| 6. | 65 Male melanoma | ZME-018 IgG/20 mg | × 2,560[b] N.D.[c] | 100 | 3 | 87 | 4 | 34 | 0 | 100 | 3 | 15 | 4 | 100 | 24 | 18 | 20 |
| 7. | 78 Male AMI | R11D10 Fab/0.5 mg | × 450[b] N.D.[c] | N.D. | | 54 | 6 | 24 | 0 | 84 | 5 | 7 | 7 | N.D. | | N.D. | |
| Normal human serum | | | | 3.7 ± 5.2[d] | | 3–8[d] | | 0[e] | | 3–9[e] | | 9–15[e] | | 13.5 ± 2.9[d] | | 9.5 ± 1.6[d] | |

[a]The level of complex formation was expressed as the percentage of counts found in complexes. PBS; phosphate buffered saline/bovine serum albumin. NMS; normal murine serum. N.D.; not done. AMI; acute myocardial infarction; [b]Fab fragment of R11D10 as immobilised Ab; [c]mixture of murine MoAbs (IgG) as immobilised Abs; [d]mean ± s.d. of eight healthy adults; [e]range of three healthy adults; [f]0% after addition of unlabelled ZCE-025 Ab.

**Figure 1** Size exclusion HPLC chromatograms of $^{111}$In-labelled ZCE-025 (IgG) (**a**,**c**,**d**) and $^{111}$In-labelled Fab fragment of anti-myosin antibody (**b**). Vertical axis shows arbitrary scale of radioactivity. **a**,**b**, $^{111}$In-labelled IgG (**a**) and $^{111}$In-labelled Fab (**b**) after incubation with normal human serum. **c**, $^{111}$In-labelled ZCE-025 after incubation with HAMA positive serum (case 4), the radioactivity was eluted in a higher molecular weight peak (Vo;void volume) than the original IgG peak. **d**, $^{111}$In-labelled ZCE-025 after incubation with HAMA positive serum (case 4) and normal murine serum. Addition of normal murine serum displaced almost all the radioactivity to the original IgG peak.

**Figure 2** Size exclusion HPLC chromatograms of $^{125}$I-labelled NL-1 and cNL-1 after incubation with HAMA positive serum (case 5). **a**,**b**, $^{125}$I-labelled NL-1 showed a higher molecular weight peak (**a**). Addition of normal murine serum displaced most of the radioactivity to the original IgG peak, but some of the radioactivity remained in the higher molecular weight peak (**b**). **c**,**d**, $^{125}$I-labelled cNL-1 formed a smaller peak than the original NL-1 (**c**), which was slightly absorbed after incubation with normal murine serum (**d**).

This immune complex formation could be inhibited by incubating with excess normal murine serum and most radioactivity was displaced to the parental IgG (Figure 1d) or Fab peak. In case 2 with colorectal cancer who had previously received 42 mg of ZCE-025 Ab, and case 5 with malignant melanoma who had previously received 20 mg of 96.5 Ab, 18% and 28% of the radioactivity was still associated with the immune complexes, respectively, even after incubation with excess normal ICR or BALB/c murine serum. However, the immune complex formation could be completely absorbed by incubating with 50 μg of unlabelled ZCE-025 Ab, but not by other anti-tumour MoAbs, indicating the presence of anti-idiotype Ab in the serum of case 2. (Case 5 was not determined due to the lack of unlabelled 96.5 Ab).

Fab fragments of murine MoAbs and chimeric human/mouse antibodies demonstrated different reactivities. Cases 2 and 3, who received whole IgG, failed to form immune complexes with R11D10 Fab as determined by HPLC analysis and ELISA assay, whereas cases 4 and 7 who were infused with $F(ab')_2$ and Fab fragments, respectively, were reactive with $^{111}$In-labelled Fab fragment as well as with all whole IgGs examined. After incubating chimeric NL-1 and SF-25 antibodies with HAMA positive serum, minimal formation of the higher molecular weight species was detectable, which was hard to displace by adding excess normal human (data not shown) or normal murine sera (Figure 2). Similar small immune complex formation was seen in normal human serum (Table I).

### Discussion

Murine MoAb infusion in humans should induce a HAMA response, which may be a key obstacle to the repeated infusion of MoAbs (Reynolds *et al.*, 1989; Dillman, 1990). To evaluate HAMA, we used ELISA and size exclusion HPLC analysis and significant relation was observed between titers assessed by the two methods. HPLC provides a simple method to evaluate changes in molecular size as would occur with the formation of radiolabelled MoAb and HAMA (Reynolds *et al.*, 1987). In our HPLC system, the 50 ng of radiolabelled MoAbs in 100 μl of HAMA positive serum is equivalent to 1.5 mg of MoAbs in 3,000 ml of plasma volume. If MoAbs exceed this quantity, the complex formation of MoAbs and HAMA decreases. Since more than 1.5 mg of MoAb is infused in most of the clinical immunoscintigraphies, our HPLC assay estimates sufficiently the degree of the *in vivo* complex formation. However, it is difficult to distinguish anti-idiotypic antibodies from circulating antigens by incubating sera with MoAbs and analysing the complex formation using HPLC. Binding of $^{111}$In-labelled ZCE-025 to the serum of case 2 with colorectal cancer was not wholly blocked by excess normal murine serum or by murine MoAbs, although excess unlabelled ZCE-025 inhibited the binding completely. The serum CEA concentrations in case 2 were within upper normal limits of less than 2.5 ng ml$^{-1}$ (serum CEA levels exceeding 100 ng ml$^{-1}$ were detectable by our HPLC system, data not shown), and a part of HAMA in this patient seemed to be reactive with the idiotype of ZCE-025 antibody (Shawler *et al.*, 1985).

Fab or $F(ab')_2$ fragments are expected to reduce the rate of developing HAMA after the injection of murine Abs. Brown *et al.* reported the absence of HAMA response in 663 patients who were given 0.5 mg of $^{111}$In-labelled anti-myosin Fab fragments (Brown *et al.*, 1988). However, in the multicenter studies performed in Japan, five out of 406 (1.2%) patients developed HAMA after a single infusion of anti-myosin Fab, determined using a similar ELISA assay (Kawai *et al.*, 1990). A positive HAMA response to the IgG and Fab was also found in case 7 with acute myocardial infarction using HPLC analysis. The difference in the incidence of HAMA in patients with heart diseases between the USA and Japan remains to be explained.

Another approach, which should reduce HAMA But utilise murine MoAb is the generation of human/mouse chimeric MoAbs (Morrison *et al.*, 1984; Brown *et al.*, 1987). Genetic technology has made possible the exchange of the mouse constant region domains with those of human, to fabricate human/mouse chimeric antibodies, which retain the murine variable region but otherwise are human (Nishimura *et al.*, 1987). By ligating the human heavy-chain enhancer element to chimeric light- and heavy-chain genes, human/mouse chimeric MoAbs were produced efficiently which reacted with a common acute lymphocytic leukaemia antigen (CALLA). Two human/mouse chimeric antibodies demonstrated little if any, reactivity with seven HAMA positive sera.

The sera of cases 4 and 7 obtained after the infusion of $F(ab')_2$ and Fab fragments, respectively, were reactive with both murine IgG and Fab, but not with the human/mouse chimeric monoclonal antibodies. It is very likely that the sera of the two patients and three out of five patients who received whole IgG and showed complex formation with R11D10 Fab, reacted with the CH1 and/or CL regions of the murine IgG. All seven HAMA positive sera bound to all radiolabelled murine MoAbs (IgG) and five out of seven patients were reactive with Fab of murine MoAb. In contrast, cNL-1 or cSF-25 MoAbs were not reactive with these HAMA positive sera. In the clinical studies using the human/mouse chimeric MoAbs, the antibody responses of the patients depend upon the infused chimeric MoAb, and the immunogenic potential of the chimeric MoAbs ranges wide (Meredith et al., 1991). The human/mouse chimeric MoAbs, especially those with low immunogenicity, are useful for repeated infusion of anti-tumour MoAbs in HAMA positive patients. Meanwhile, excess normal murine serum did not completely displace the binding of NL-1 or SF-25 MoAbs to HAMA positive serum and up to 15 to 24% of radioactivity was observed in a large molecular weight peak even after incubating with HAMA negative normal human serum. NL-1 recognises common acute lymphocytic leukaemia antigen (CALLA), which is identical with neutral endopeptidase (Letarte et al., 1988; Monod et al., 1989), and is clinically employed as an important cell surface marker for the diagnosis of human acute lymphocytic leukaemia. However, CALLA is not restricted to leukaemic cells and is also found on a variety of normal tissues (Losa et al., 1986). Complexes formed after adding normal murine serum or when incubated with normal human serum are most likely due to the binding of circulating antigens with radiolabelled MoAbs.

In summary, (1) HAMA of all seven patients formed complexes with all four murine IgGs, but not with two human/mouse chimeric antibodies, (2) five out of seven patients showed reactivity with R111D10 Fab in their sera, suggesting that they have HAMA recognising CH1 or CL region, (3) one patient seemed to have anti-idiotypic antibody. HAMA showed variety in reactivity with murine MoAbs but human/mouse chimeric antibodies are promising even in most patients who became HAMA positive after repeated infusion with murine MoAbs.

The authors are grateful to Centocor and Toray Industries, Inc. for the supply of SF-25 and cSF-25 antibodies.

### References

ABDEL-NABI, H.H., SCHWARTZ, A.N., HIGANO, C.S., WECHTER, D.G. & UNGER, M.W. (1987). Colorectal carcinoma: detection with indium-111 anticarcinoembryonic-antigen monoclonal antibody ZCE-025. *Radiology*, **164**, 617.

BROWN, B.A., DAVIS, G.L., SALTZGABER-MULLER, J. & 6 others (1987). Tumor-specific genetically engineered murine/human chimeric monoclonal antibody. *Cancer Res.*, **47**, 3577.

BROWN, J.M., DEAN, R.T., KAPLAN, P. & 4 others (1988). Absence of human antimouse antibody (HAMA) response in patients given antimyosin Fab-DTPA monoclonal antibody. *J. Nucl. Med.*, **29**, 851.

CHATAL, J.F., SACCAVINI, J.C., FUMOLEAU, P. & 5 others (1984). Immunoscintigraphy of colon carcinoma. *J. Nucl. Med.*, **25**, 307.

COCHLER, D., MILENIC, D.E., FERRONI, P. & 5 others (1990). *In vivo* fate of monoclonal antibody B72.3 in patients with colorectal cancer. *J. Nucl. Med.*, **31**, 1133.

CORTENAY-LUCK, N.S., EPENETOS, A.A., MOORE, R. & 4 others (1986). Development of primary and secondary responses to mouse monoclonal antibodies used in the diagnosis and therapy of malignant neoplasms. *Cancer Res.*, **46**, 6489.

DILLMAN, R.O. (1990). Human antimouse and antiglobulin responses to monoclonal antibodies. *Antibody, Immunoconjugates, and Radiopharmaceuticals*, **3**, 1.

GREENWOOD, F.C., HUNTER, W.M. & GLOVER, J.S. (1963). The preparation of $^{131}$I-labelled human growth hormone of high specific radioactivity. *Biochem. J.*, **89**, 114.

HUNTER, W.M. & GREENWOOD, F.C. (1962). Preparation of iodine-131 labeled human growth hormone of high specific activity. *Nature*, **194**, 495.

KAWAI, C., MATSUMORI, A., NISHIMURA, T. & ENDO, K. (1990). $^{111}$I-antimyosin Fab scintigraphy in cardiovascular diseases: multicenter clinical trial. *Jpn. J. Nucl. Med.*, **27**, 1419.

KHAW, B.A., MATTIS, J.A., MELINCOFF, G., STRAUSS, H.W, GOLD, H.K. & HABER, E. (1984). Monoclonal antibody to cardiac myosin. *Hybridoma*, **3**, 11.

KHAW, B.A., YASUDA, T., GOLD, H.K. & 6 others (1987). Acute myocardial infarct imaging with $^{111}$In-labeled monoclonal antimyosin Fab. *J Nucl. Med.*, **28**, 1671.

KOIZUMI, M., ENDO, K., WATANABE, Y. & 7 others (1988). Immunoscintigraphy and pharmacokinetics of indium-111-labeled ZEM-018 monoclonal antibody in patients with malignant melanoma. *Jpn. J. Cancer Res.*, **79**, 973.

LETARTE, M., VERA, S., TRAN, R. & 5 others (1988). Common acute lymphocytic leukemia antigen is identical to neutral endopeptidase. *J. Exp. Med.*, **168**, 1247.

LOSA, G.A., HEUMANN, D., CARREL, S., FLIENDNER, V.V. & MACH, J.P. (1986). Characterization of membrane vesicles circulating in the serum of patients with common acute lymphoblastic leukemia. *Lab. Invest.*, **55**, 573.

MEREDITH, R.F., LOBUGLIO, A.F., PLOTT, W.E. & 13 others (1991). Pharmacokinetics, immune response, and biodistribution of iodine-131-labeled chimeric mouse/human IgG1, k 17–1A monoclonal antibody. *J. Nucl. Med.*, **32**, 1162.

MONOD, L., DISERENS, A.C., JONGENEEL, C.V. & 4 others (1989). Human glioma cell lines expressing the common acute lymphoblastic leukemia antigen (cALLa) have neutral endopeptidase activity. *Int. J. Cancer*, **44**, 948.

MORRISON, S.L., JOHNSON, M.L., HERZENBERG, L.A. & OI, T.W. (1984). Chimeric human antibody molecules: mouse antigen-binding domains with human constant region domains. *Proc. Natl Acad. Sci. USA*, **79**, 4386.

NISHIMURA, Y., YOKOYAMA, M., ARAKI, K., UEDA, R., KUDO, A. & WATANABE, T. (1987). Recombinant human-mouse chimeric monoclonal antibody specific for common acute lymphocytic leukemia antigen. *Cancer Res.*, **47**, 999.

REYNOLDS, J.C., VECCHIO, S.D., LORA, M.E., CARRASQUILLO, J.A. & LARSON, S.M. (1987). Antibody-antibody complexes are related to human anti-murine antibody (HAMA). *Nuklearmedizin Suppl.*, **27**, 555.

REYNOLDS, J.C., VECCHIO, S.D., SAKAHARA, H. & 4 others (1989). Anti-murine antibody response to mouse monoclonal antibodies: clinical findings and implications. *Int. J. Rad. Appl. Instum [B].*, **16**, 121.

SAGA, T., ENDO, K., KOIZUMI, M. & 7 others (1990). *In vitro* and *in vivo* properties of human/mouse chimeric monoclonal antibody specific for common acute lymphocytic leukemia antigen. *J. Nucl. Med.*, **31**, 1077.

SAKAHARA, H., ENDO, K., NAKASHIMA, T. & 9 others (1985). Effect of DTPA conjugation on the binding activity and biodistribution of monoclonal antibodies against alpha-fetoprotein. *J. Nucl. Med.*, **26**, 750.

SAKAHARA, H., REYNOLDS, J.C., CARRASQUILLO, J.A. & 5 others (1989). *In vitro* complex formation and biodistribution of mouse antitumor monoclonal antibody in cancer patients. *J. Nucl. Med.*, **30**, 1311.

SCHROFF, R.W., FOON, K.A., BEATTY, S.M., OLDHAM, R.K. & MORGAN, A.C. Jr. (1985). Human anti-murine immunoglobulin responses in patients receiving monoclonal antibody therapy. *Cancer Res.*, **45**, 879.

SHAWLER, D.L., BARTHOLOMEW, R.M., SMITH, L.M. & DILLMAN, R.O. (1985). Human immune response to multiple injections of murine monoclonal IgG. *J. Immunol.*, **135**, 1530.

TAKAHASHI, H., WILSON, B., OZTURK, M. & 4 others (1988). *In vivo* localization of human colon adenocarcinoma by monoclonal antibody binding to a highly expressed cell surface antigen. *Cancer Res.*, **48**, 6573.